# Extending the “Social”: Anthropological Contributions to the Study of Viral Haemorrhagic Fevers

**DOI:** 10.1371/journal.pntd.0003651

**Published:** 2015-04-16

**Authors:** Hannah Brown, Ann H. Kelly, Almudena Marí Sáez, Elisabeth Fichet-Calvet, Rashid Ansumana, Jesse Bonwitt, N’Faly Magassouba, Foday Sahr, Matthias Borchert

**Affiliations:** 1 Anthropology Department, Durham University, Durham, United Kingdom; 2 Department of Sociology, Philosophy and Anthropology, Exeter University, Exeter, United Kingdom; 3 Department of Global Health and Development, London School of Hygiene and Tropical Medicine, London, United Kingdom; 4 Institute of Tropical Medicine and International Health, Charité—Universitätsmedizin Berlin, Berlin, Germany; 5 Department of Virology, Bernhard Nocht Institute for Tropical Medicine, Hamburg, Germany; 6 Mercy Hospital Research Laboratory, Bo, Sierra Leone; 7 Infectious and Tropical Diseases Department, National Hospital Donka, Conakry, Guinea; 8 Department of Microbiology, College of Medicine and Allied Health Sciences, University of Sierra Leone, Freetown, Sierra Leone; 9 Department of Infectious Disease Epidemiology, London School of Hygiene and Tropical Medicine, London, United Kingdom; University of Queensland, AUSTRALIA

## Anthropology and the One-Health Agenda for VHF

Emerging Viral Haemorrhagic Fevers (VHFs) offer a frontier for a “One-Health” research agenda; the joined-up, or collaborative, effort of multiple disciplines to attain optimal health for people, animals, and the environment (e.g., http://www.onehealthinitiative.com/). Our multidisciplinary work on Lassa fever and Ebola Virus Disease in Guinea and Sierra Leone explores the connections between humans, rodents such as the *Mastomys natalensis* (Natal multimammate mouse), and the broader environmental conditions that facilitate virus transmission. In this viewpoint, we outline our vision for an anthropological contribution to the study of VHFs.

Research into the control and emergence of VHFs has been characterised by an interdisciplinary approach [1]. While anthropologists have formed a critical part of outbreak response [2–5], we see greater scope for anthropological work on primary and secondary routes of infection. Existing anthropological studies of human-to-human transmission of VHF have focused predominately upon “local beliefs” such as funeral rites and traditional healing practices. For example, in Gulu, Northern Uganda, the Hewletts and their colleagues have documented how indigenous Acholi understandings of epidemics as caused by a bad spirit (*gemo*) involved responses that curtailed the spread of Ebola and supported public health efforts [2,3]. Epelboin [5] has also worked with outbreak teams in a number of sites to improve interventions, for example, by developing locally appropriate but safe modes of greeting and burial.

These studies have not only succeeded in improving the effectiveness of VHF management but also in normalising the inclusion of anthropological perspectives in public health interventions during an epidemic [6]. However, while understanding local beliefs and customary practices provides significant insight into VHFs and how we should respond to outbreaks, it confines the anthropological contribution to an unnecessarily narrow remit. To understand the dynamics of VHF transmission demands finely-grained attention to localised social practices and the objects and places that shape everyday life [7]. We argue for an expansion of what are considered to constitute the “social,” or cultural, factors of VHF to build upon a growing anthropological interest in interactions between humans and animals and the ways in which people relate to, use, and live with objects. These approaches offer fresh insights into the movement of pathogens from the bush and gardens to homes and hospital, extending knowledge about both primary and secondary transmission dynamics [8]. This reconceptualisation of the “social” provides a new and important entry point for further development of a One-Health Agenda by bringing anthropological insights into conversation with the concerns of ecologists and epidemiologists. Our viewpoint offers examples of our work on primary and secondary VHF transmission that demonstrate how an anthropological attention to an expanded social field can enhance knowledge about mechanisms of disease emergence, spread, and amplification. Such insights may prove useful to those who seek to improve interventions to reduce VHF transmission or wish to make disease containment interventions more acceptable to affected communities.

## The Social Lives and Spaces of Animals

The recent surge of interest in the significance of animals to social life in anthropology [9–11] provides a valuable entry point for thinking about interspecies transmission. This work recognises that humans do not simply relate to animals as sources of sustenance or as symbols of the natural world, but that animals and humans are engaged in “social” relationships, which are created through the actions of both parties. For instance, anthropological work on the economies, practices, and symbolic resonances of hunting has relevance for how humans come into contact with the hosts of the Ebola, Lassa, and Marburg viruses [12]. Extending these concerns with processes of commensality can shed considerable light on how occasions for pathogenic exchange between species arise.

Emerging data from our anthropological work on Lassa underlines the importance of studying the social constitution of domestic spaces in order to develop insights around the ways that *M*. *natalensis* and humans share homes. The material organisation of homes creates many possibilities for risky contact, including through food storage practices, the use of everyday domestic objects, and sleeping or resting arrangements. An association with the domestic realm appears to render *M*. *natalensis* a “friendly,” although irritating “housemate,” in contrast to the “wild” animals of the “bush.” Despite their noise and forays into food stores, *M*. *natalensis* are considered a normal feature of domestic life. The material and symbolic dimensions of domestic spaces shape opportunities for disease transmission in ways that are compounded by socioeconomic constraints. In Lassa-affected villages in Sierra Leone, people have described being urinated upon by rats when sleeping and claim they cannot always afford to throw away cooked food that rats have come into contact with [13].

Our work also shows the value of anthropological methods for differentiating hunting practices that may facilitate primary transmission. The involvement of Marí Sáez (an anthropologist) in interdisciplinary investigations into the zoonotic origins of the Ebola outbreak in Guinea helped draw attention to the potential significance of children’s hunting practices in this transmission event [14]. Meanwhile, the hunting of rodents, i.e., *Mastomys* spp., *Praomys* spp., and *Lophuromys sikapusi* in Sierra Leone is sometimes done through purposeful trapping but is also routinised as an opportunistic side effect of domestic agriculture, producing different forms of contact between rodents and humans [13]. The public health application of this kind of data is 2-fold—firstly, data of this kind provide a rich picture of the shared social worlds of humans and animals and forms of contact that may spread disease. Secondly, such insights can be used to develop locally acceptable interventions to reduce primary transmission by encouraging safer forms of hunting and butchering meat from wild animals rather than reactive bans which may be ineffective in places where people have eaten such animals for many years, rely heavily upon them for protein and taste, and do not understand the rationale behind such a ban.

## The Outbreak Ward: Social Objects, Social Spaces

Understanding human amplification requires a different repertoire of empirical resources from those necessary to investigate the dynamics of primary transmission. VHF outbreaks in Africa bring an influx of disease managers, volunteers, and clinicians. These teams introduce rapid disease control interventions structured by enormous inequities of resource availability. Their work may involve forming infection control fences from logs and sticks or isolation wards from tents or containers. Corpses of loved ones are buried by people trained to follow strict protocol for donning and doffing the strange garb of full body protection; bedding and other objects that have come in contact with the sick are burnt (see, for example, Alain Epelboin’s film, *Ebola au Congo* http://www.pathexo.fr/docfiles/ebola-congo-1.html). These reorganisations of spatial and material worlds are among the most striking aspects of interventions to manage Ebola and other VHF outbreaks, yet their significance for transmission has not drawn the sustained attention of anthropologists and other social scientists.

Much equipment used in outbreaks carries diverse understandings of detachment, contact, visibility, and contagion. Take the example of protective clothing: standard and ubiquitous tools of barrier nursing, such as gloves, are involved in—and shape—many different kinds of social relationships. For example, health workers providing care during a Marburg outbreak in the Democratic Republic of Congo failed to properly use protective gear and cases of occupational infection continued to arise. These health workers were trained in barrier nursing and provided with supplies donated by international organisations. However, they sometimes wore gloves for long periods of time without disinfecting their gloved hands or changing them regularly to prevent them from becoming porous in the heat. The same health workers also reported that the use of protective clothing could demonstrate detachment and fear in a context where “proper” care required the intimacy of human touch, particularly when treating colleagues or family members. Indeed, many patients expressed preference in being treated by health workers without gloves, as the use of gloves suggested that the patient was “dirty.” It is clear that making sense of the multiple meanings, uses, and social capacities of gloves and other protective objects is key to improving their use during outbreaks [15].

## Conclusion

An extended “social” lens suggests new sites at which anthropologists might usefully contribute to VHF research, prevention, and control. Existing anthropological work on VHFs has largely focused on how to modify outbreak control efforts to make them more acceptable to the local population and how to decrease the risk of secondary transmission. This line of inquiry is very important indeed. These insights can be further developed through ethnographic attention to hospital spaces, materials, and practices, and how these are used by both local populations and public health teams. In this way, anthropologists can deepen understandings of the social dimensions of care, the lasting impact of epidemics, and the efforts to control them.

This extended notion of the “social” draws attention to the social relations between humans, material objects, and animal hosts that open up pathways of transmission, offering greater purchase upon both primary transmission from animal populations to humans and secondary transmission between humans. Our approach underlines sites at which anthropological insights could drive a future research agenda for VHF. It also marks out the possibility for an extended and newly productive engagement between anthropologists, disease ecologists, epidemiologists, and disease control specialists and for a richer and more nuanced understanding of VHFs themselves.
