# Correction: Extending the "Social": Anthropological Contributions to the Study of Viral Haemorrhagic Fevers

**DOI:** 10.1371/journal.pntd.0003775

**Published:** 2015-05-07

**Authors:** The PLOS Neglected Tropical Diseases Staff

Two grant numbers are inadvertently omitted in the Funding section. The correct funding information is as follows:

Authors are funded by the following grants from the DFG (http://www.dfg.de/en/) (DFG BO3790/1-1), DFG (FI1781/1-1) and the following grants from the ESRC (http://www.esrc.ac.uk/) (ES/L010690/1) and (ES/M009203/1). The funders had no role in study design, data collection and analysis, decision to publish, or preparation of the manuscript.

The publisher apologizes for this error.
